# Lived experiences of LGBTQI+ persons when accessing primary healthcare facilities in Gauteng, South Africa: An interpretative phenomenological inquiry

**DOI:** 10.4102/curationis.v49i1.2838

**Published:** 2026-08-19

**Authors:** George J. Nkabinde-Thamae, Charlene Downing

**Affiliations:** 1 Department of Nursing, Faculty of Health Sciences, University of Johannesburg, Johannesburg, South Africa

**Keywords:** LGBTQI+, discrimination, homophobia, healthcare, primary healthcare, inclusivity

## Abstract

**Background:**

Access to dignified healthcare services is regarded as a human right; however, individuals identified as LGBTQI+ persons still appear to be humiliated and uncared for when accessing primary healthcare facilities.

**Objectives:**

This study explored and described the experiences of the LGBTQI+ persons when accessing primary healthcare facilities.

**Method:**

A qualitative interpretative phenomenological analysis was employed. Unstructured individual interviews were conducted with 16 self-identified LGBTQI+ persons from five districts and analysed using inductive thematic analysis.

**Results:**

Three themes were identified: (1) social injustice, (2) professional nurses’ unpreparedness for inclusive care and (3) experiences of affirmative care. Each theme encompassed relevant sub-themes.

**Conclusion:**

The results revealed a lack of humanity and uncaring healthcare services that LGBTQI+ individuals received when accessing primary healthcare facilities in Gauteng, South Africa.

**Contribution:**

These findings advocate for inclusive, respectful, dignified and sensitive healthcare services.

## Introduction

Gender and sexually diverse individuals (collectively also referred to as LGBTQI+) have various health needs, which are frequently unmet in primary healthcare facilities (Javier 2024; Wahlen et al. 2020). Their varied healthcare needs often relate to sexual orientation, gender identity and sexual development, areas that are not prioritised within medical and nursing training, resulting in health disparities, discrimination and homophobic attacks (Katz-Wise et al. 2023). The lived experiences of LGBTQI+ persons in primary healthcare facilities in Gauteng are explicitly unveiled in an article, ‘God is against you guys’. The key findings of this article indicate that these individuals continue to encounter stigma, discrimination and even denial of healthcare services in these healthcare facilities (Igual 2023). Gwala (2026) shares that a lack of an affirming healthcare environment, socioeconomic vulnerability and a strained public healthcare worsens the experiences of LGBTQI+ persons in primary healthcare facilities. Schmitz and Tabler (2021) note that previous negative experiences, such as stigma, discrimination and healthcare workers’ judgement towards LGBTQI+ persons, justified their decisions not to access healthcare facilities despite their healthcare needs.

Similar findings were observed in a study conducted in Lebanon, a small country in the Middle East, where LGBTQI+ persons often delay accessing healthcare facilities, which stems from continuous negative experiences in healthcare facilities (Daoud et al. 2025). LGBTQI+ persons often fear accessing healthcare institutions, which emanates from the fear of mistreatment by healthcare workers (Chedid et al. 2024). These experiences are related to a heteronormative healthcare environment that appears to be only empowered to provide healthcare services for those who identify as heterosexual (Adley, O’Donnell & Scott 2025).

Further, LGBTQI+ persons seem to experience a lack of healthcare services that are inclusive or affirming, such as lubrication, finger gels and even unisex toilets to create a nonbinary healthcare environment, to meet their individualised healthcare needs (Huff et al. 2023). Within the context of primary healthcare facilities, access means having access to the healthcare services an individual needs, not merely to the physical building (Arrol & Goodyear-Smith 2024; World Health Organization [WHO] 2024). Based on the literature above and drawing on the definition of access, it appears that the various barriers, namely personal (individual) and systemic, have a direct impact on their health outcomes (Mkhize & Maharaj 2023). The literature indicates that those who identify as gay men are at risk for HIV (human immunodeficiency virus) and syphilis - conditions that are often left untreated and affect their general well-being (McNamara & Ng 2016; Tran & Nguyen 2024). Further limited access to healthcare facilities deprives LGBTQI+ persons of quality health services, which predisposes them to other health conditions, such as heart disease and other chronic conditions like asthma and hypertension (Kasprowski et al. 2021). In addition, Haviland et al. (2020) state that cancer diseases (prostate and cervical cancers) remain a health threat to all human beings and an even greater risk for LGBTQI+ individuals, as such screenings for LGBTQI+ persons are often not prioritised by healthcare organisations. As a result of a heteronormative approach to sexual and reproductive healthcare, self-identified lesbian women are often neglected in routine cervical screening (Yu, Bauermeister & Flores 2023). In addition, the literature unveils that even individuals who self-identify as either transgender women or men are not prioritised with periodic screening (Carroll et al. 2023; Gibson et al. 2022). The compromised healthcare services LGBTQI+ people experience in primary healthcare facilities affect their mental well-being, as evidenced by anxiety, depression and even suicide incidents, among these individuals (Izutsu &Tsutsumi 2024). LGBTQI+ persons are often classified as a marginalised community that has been significantly excluded from healthcare, relevant health policies and patient-centred research, which contributes towards their healthcare needs not being prioritised (Sachdeva et al. 2021). The literature further unveils that the healthcare disparities LGBTQI+ persons experience in primary healthcare make them feel disrespected and humiliated, resulting in them feeling uncared for and dissatisfied with the care they received when accessing these healthcare facilities (Storino & Barrios 2023).

Hackbart et al. (2024) argue that, despite efforts made nationally and internationally, LGBTQI+ persons continue to experience fragmented healthcare and discrimination when accessing healthcare services. Similarly, Lessard, Watson and Puhl (2020) state that LGBTQI+ people can only receive comprehensive, quality care when healthcare workers understand the healthcare needs of these individuals and provide care that’s free from discrimination and judgment. The literature above provides clear insight into healthcare facilities’ experiences; however, little is known about their lived experiences when accessing primary healthcare facilities in Gauteng, South Africa (Gonçalves et al. 2024). Thus, the researchers believe that despite the phenomenon of LGBTQI+ people being explored extensively in other related research, this specific context and focus will promote awareness of the experiences of LGBTQI+ persons in Gauteng, South Africa, and interventions that are needed to promote inclusive, respectful, dignified and sensitive healthcare services with primary healthcare facilities.

### Problem statement

LGBTQI+ persons globally experience healthcare challenges and dissatisfaction with the care they receive in primary healthcare facilities, as evidenced by the constant discrimination, homophobia and stereotypical attitudes from professional nurses (Medina-Martínez et al. 2021). The primary researcher, a senior manager employed in a primary healthcare environment overseeing primary healthcare services, often receives verbal or written complaints relating to discrimination, homophobia and discriminatory attitudes from self-identified LGBTQI+ persons when they access primary healthcare facilities. This prompted a scientific inquiry into understanding what contributes to such experiences, as primary healthcare services should be accessible to everyone, irrespective of race, sex, gender or social standing. This statement is supported by the National Department of Health, which reiterates that accessible healthcare services should be provided to everyone who needs them and without discrimination (www.national department of health 2021). Dahir (2024) affirms that despite the positive commitments various countries made to develop and implement policies to promote inclusive healthcare services, LGBTQI+ individuals still experience health disparities.

Primary healthcare facilities appear to be only providing care to those who are classified as heterosexual, which promotes the experiences of discrimination, stigma and uncaring moments by LGBTQI+ people (McGowan, Lowther & Meads 2021). Various literature concurs that the challenges LGBTQI+ persons experience in primary healthcare facilities are contributed to by a healthcare system that is designed for a heteronormative society, which automatically excludes those who identify as LGBTQI+ individuals (Dube et al. 2026; Ramalepe & Maake 2025). A systematic review study in Canada found that LGBTQI+ people experienced longer waiting times when seeking healthcare services, which often results in them delaying or avoiding seeking medical care (Ansell et al. 2017). Further, Moyo, Macherera and Mavhandu-Mudzusi (2021) unveiled that men having sex with other men in Zimbabwe often experience stigmas and discrimination when accessing primary healthcare facilities, in comparison to heterosexual males. Specifically to the South African context, the lack of resources (lubricants, finger gels, hormonal tablets), non-inclusive environments and clear policies containing content for the LGBTQI+ persons contribute to an unfavourable experience of care in primary healthcare facilities (Sefolosha, Van Wyk & Van der Wath 2021). Subsequently, the WHO has noted that LGBTQI+ persons experience health disparities, especially those who are identified as transgender, and concluded the need for the development of guidelines that promote inclusion and how to prevent stigma and discrimination among LGBTQI+ people (Rosa et al. 2024). The literature and narrative above give insight into the challenges and realities that LGBTQI+ persons encounter globally when accessing primary healthcare facilities.

### Theoretical framework

This study was underpinned by intersectionality theory. The researchers found this theory applicable to this study, as it focuses on components such as oppression, marginalised communities and discrimination (Liu 2023). Moreover, this theory provides a lens into different aspects of an individual’s identity, including class, race, sexuality and nationality (Tracy 2025). Intersectional theory addresses systemic barriers experienced by LGBTQI+ persons (Sekoni, Jolly & Gale 2022). Specific to this study, this theory explored the unbearable experiences encountered by LGBTQI+ persons in primary healthcare facilities and the unpreparedness of professional nurses in providing care to LGBTQI+ individuals. Kline (2022) advises that applying intersectional theory effectively will assist professional nurses and other health professionals in recognising the specific healthcare needs of LGBTQI+ people and in providing mindfulness to LGBTQI+ persons, enabling healthcare workers to provide individualised healthcare.

#### Purpose

The study explored the lived experiences of LGBTQI+ persons when accessing primary healthcare services in Gauteng, South Africa.

## Research methods and design

### Study design

This study employed an interpretive phenomenological analysis to explore in depth how self-identified LGBTQI+ persons experienced primary healthcare services when accessing these facilities for healthcare services. Interpretive phenomenological analysis appeared relevant for this study as the researchers were not only interested in exploring the participants’ experiences in primary healthcare facilities but also to understand what these experiences meant to them and their interpretation thereof (Smith & Fieldsend 2021). This method was deeply rooted in Heidegger’s hermeneutic phenomenology, which believes that human beings can interpret their own experiences based on their individual experiences (Cooke et al. 2026). Within this study, this method promoted a double hermeneutic, which facilitated meaningful dialogue between the 16 participants understanding, of their own experiences within primary healthcare facilities in Gauteng, and the researchers own interpretative lens as researchers, which assisted the researchers not only to understand what happened but also to understand the meaning of the participants experiences, in order to have transformative insight (Alsaigh & Coyne 2021, Wanat, Day & Larkin 2025).

### Population and sampling

The population in this study comprised self-identified LGBTQI+ persons accessing public healthcare facilities in the Gauteng province. From the population, 16 (*N* = 16) participants consented to participate in the study through a non-probability snowball sampling technique. This method was employed because these participants are regarded as a hard-to-reach community that is often marginalised, thus using a peer network (Nkabinde-Thamae & Downing 2026; Tukisi et al. 2025).

### Recruitment of participants

The participants were recruited from primary healthcare facilities in Gauteng, South Africa, from all five districts (City of Ekurhuleni, Tshwane, Sedibeng, West Rand and the City of Johannesburg). The primary researcher shared a poster on social media platforms, such as Facebook, which contained all the relevant information about the study, the title of the research study, aims and objectives, inclusion and exclusion criteria, emphasis on the ethical principles and the primary researcher’s contact details for participants seeking clarification or expressing interest in participating in the study. To ensure that even individuals not on social media were reached, the primary researcher hand-delivered flyers at the approved facilities. Potential participants used the primary researcher’s contact details for clarification and to express their interest in participating. Participants were considered enrolled only after attending the information session and signing the informed consent.

### Research setting

This study was conducted in primary healthcare facilities across the entire Gauteng province (City of Ekurhuleni, City of Johannesburg, Sedibeng, Tshwane and West Rand). These are fixed healthcare facilities in urban areas, led by my multidisciplinary team of healthcare workers, comprising primary healthcare nurses and medical doctors, operating Monday to Friday, 08:00 am-16:30 pm (Mashazi et al. 2025; Seretlo & Mokgatle 2022). Moreover, these facilities provide healthcare services such as Antenatal Care (ANC), postnatal care (PNC), immunisations and chronic disease management (Kapologwe et al. 2020).

### Inclusion criteria

Specific inclusion criteria were used to ensure that relevant participants were recruited, as advised by Keung et al. (2020). Thus, the *inclusion criteria* for this study were as follows:

- The potential participants had to self-identify as LGBTQI+.
- The potential participants had to reside within Gauteng province to ensure the research was contextual.
- The potential participants had to have attended a PHC clinic in the last 6 months on more than two occasions, which would allow them to share their current lived experiences when accessing a primary healthcare facility in Gauteng, South Africa.
- The potential participants had to be 18 years or older to consent, where needed.

### Data collection

Data collection commenced upon receipt of all relevant ethical clearance and gateway approvals. Data collection occurred from June 2023 to November 2023 (6 months). Through face-to-face, unstructured, individual interviews at a place and time convenient for the participants, away from primary healthcare facilities, but rather at community centres or other public places such as restaurants, where noise was minimal, and the possibility of interruption was low. Unstructured individual interviews help researchers collect high-quality data (Tukisi, Matshidza & Malesela 2025). Unstructured interviews were employed so that the sixteen participants could share their lived experiences and be conducted in any format they felt comfortable with (Barnard, Smith & Long 2026). This format promoted the researchers’ reflexive bracketing by ensuring that interviews were focused on the participants’ lived experiences rather than those of the researchers (Smith, Larkin & Flowers 2021). Wanat et al. (2025) note that unstructured interviews are embedded in open-ended questions, which assist researchers to gain a deeper understanding and meaning of the phenomena, which provides the depth as advised by interpretative phenomenological inquiry (Aveyard et al. 2024).

The primary researcher conducted all the interviews; the secondary researcher had a supervisory role, listening to all recorded interviews to assess the authenticity of the recordings and the relevance of the collected data. All participants were asked one central question: ‘What are your experiences with the care you receive when accessing primary healthcare facilities in Gauteng, South Africa?’ This central question allowed self-identified LGBTQI+ persons who access PHC facilities as a point of care to share their lived experiences. In addition to the central question, the primary researcher posed various probing questions that emerged from participants’ responses to ensure the phenomenon was thoroughly explored and that the researchers could understand it from the participants’ perspective (Online Appendix 1, Table 1-A1). All sixteen interviews were conducted in English, lasted approximately 45–60 min and were audio recorded. In this study, interviews were conducted until data saturation was reached. Thus, data saturation was used to determine the sample size when additional participants could no longer provide further information on the phenomenon (Al-Otaibi et al. 2024). Through the individual interviews of the 16 participants across Gauteng province, the researchers determined that interpretative redundancy was achieved, resulting in the data being regarded as a thick, in-depth description of self-identified LGBTQI+ persons’ experiences in primary healthcare facilities in Gauteng, South Africa.

### Reflexive bracketing

Within this study, the researcher used reflexivity and bracketing to maintain the reliability of the study and to prevent any personal biases from influencing the data (Patton 2015). Further to validate the results of this study, multiple sources, namely audio recordings of all participants, field notes and observation notes, were kept by the researchers, which assisted in identifying and bracketing any biased experiences of the phenomena (Kawar et al. 2024). Further reflexive bracketing was employed to uphold the trustworthiness of the study (LoBiondo-Wood & Haber 2026). The data were obtained by the primary researcher, a PhD candidate with adequate knowledge and skills to conduct individual face-to-face interviews. All the auto-taped interviews were listened to by the secondary researcher, a full professor, to ensure the data collection process was authentic and from the participants’ perspective. In addition, the primary researcher became mindful that his professional background could affect the study’s analytical process; thus, reflexive bracketing was employed, with the primary researcher consciously identifying possible preconceived ideas, theories or biasness regarding the experiences of LGBTQI+ persons when accessing primary healthcare facilities in Gauteng, South Africa. Moreover, the primary researcher used a reflexive journal to continuously document any internal biases or emotional reactions to ensure the authenticity of the data collection and analysis. This allowed the themes and sub-themes to form a clear narrative of the participant’s experience rather than the primary researcher’s (Ybarra & Stephens 2025).

### Data analysis

In qualitative research, data analysis aims to reduce the volume of collected data by coding relationships and patterns in the findings to provide the researcher with a comprehensive snapshot of the data (Bush & Amechi 2019; Johnson & Christensen 2024). The researchers applied Braun and Clarke’s six steps (phases) of reflexive thematic analysis (Braun & Clarke 2019, 2022, 2023). Adopting a relativist ontological position guided the researchers to be mindful that there is no single objectivity on self-identified persons’ experiences in primary healthcare facilities, but rather a subjective and multiple one, which during the analysis process gave the researchers insight into how the 16 participants constructed meaning from their own lived experiences. The coding was done manually by the researchers: (1) The researchers engaged and familiarised themselves with the collected data by reading the field notes and listening to the audio recordings several times to understand and derive meaningful interpretations. During this process, bracketing was applied. (2) Thereafter, the researchers focused on segments of meaning that gave insight into the ‘what’ and ‘how’ of the 16 participants’ lived experiences when accessing primary healthcare facilities in Gauteng, South Africa. (3) Thereafter, the researchers determined the different patterns that appear to be true for the LGBTQI+ persons (the participants). During this stage, the researchers reviewed the themes and sub-themes against the raw data from all 16 participants. This involved looking beyond simple positive or negative binary categorisation to identify contradictions and fine details. This process ensured that the themes and sub-themes provided a comprehensive and nuanced account of the participants’ experiences in Gauteng healthcare facilities. (4) During this step, reflexive bracketing was upheld, as the researchers constantly compared the sixteen transcripts, resulting in the themes and sub-themes being captured to present a deeper interpretative meaning of the participants’ own lived experiences. (5) Within this study, the themes and sub-themes were defined to be inclusive of the participants’ verbal contributions to the phenomenon and any underlying emotional nuances, which assisted the researchers to develop a richer understanding of the participants. Lastly, a detailed report was generated, providing interpretative depth by incorporating verbatim quotes, allowing the findings to be grounded in the experiences of the 16 participants (Braun & Clarke 2022, 2023).

### Trustworthiness

Denzin and Giardina (2024) state that researchers play an imperative role within a research process; thus, in this study, the researchers were constantly aware of their own personal and interpersonal reflexivity to avoid bias or preconceived assumptions regarding this phenomenon. The primary researcher used a reflective diary to write down his daily thoughts and feelings (Silverio et al. 2022). Furthermore, trustworthiness was promoted through credibility, transferability, dependability and confirmability as advised by Lincoln and Guba (1965). Credibility was established through prolonged engagement (6 months) with the participants until data saturation was reached. An independent coder with a PhD in nursing and extensive experience in qualitative analysis conducted a secondary data review. The secondary researcher, a full professor at an esteemed university, served as the study’s supervisor. The dependability of this study was upheld through the selection process of participants, data collection and data analysis. The researchers promoted confirmability by remaining objective throughout the research process and by bracketing their own perceptions of the lived experiences of LGBTQI+ persons when accessing primary healthcare facilities in Gauteng, South Africa. Lastly, transferability was ensured by the researchers’ clear description of the study and the sampling methods.

### Ethical considerations

Before the researchers began data collection, the primary researcher held an information session with potential individual participants to provide study details and allow them to ask clarifying questions. Afterwards, participants who met the inclusion criteria were asked to provide their consent to participate. However, the primary researcher informed participants that they had the right to withdraw at any stage of the research process. Confidentiality, privacy, justice and anonymity were maintained throughout the research process (Mulaudzi & Downing 2025). Moreover, this study was subject to ethical review from the University of Johannesburg Ethics Committee (REC-1822-2022). In addition to the ethical review process, permission had to be obtained from all the districts designated as the research settings for this study.

## Results

The sample of individuals who self-identified as LGBTQI+ consisted of 16 individuals aged 23 years to 45 years. They all accessed primary healthcare facilities for various healthcare services (curative, chronic and reproductive) within the Gauteng province. A detailed summary of participants’ demographic characteristics is presented in Online Appendix 1, Table 2-A1.

### Central story

The thematic analysis revealed that self-identified LGBTQI+ persons experienced inhumane and discriminatory treatment in primary healthcare facilities in Gauteng, South Africa. Professional nurses’ bias often overshadowed ethical care, leaving participants feeling dehumanised and fearful of seeking healthcare services.

### Themes

The thematic exploration of the experiences of LGBTQI+ at primary healthcare facilities in Gauteng, South Africa, yielded three major themes, namely: (1) Social injustice, (2) professional nurses’ unpreparedness for inclusive care and (3) experiences of affirmative care. These three central themes include sub-themes as summarised in Table 1.

**TABLE 1:** Themes and sub-themes.

Themes	Sub-themes
Theme 1: Social injustice	1.1 Human rights violations 1.2 Discrimination 1.3 Compromised confidentiality
Theme 2: Professional nurses’ unpreparedness for inclusive care	2.1 Inadequate knowledge, skills and values 2.2 Imposing heterosexual norms and values 2.3 Lack of humanity
Theme 3: Experiences of affirmative care	3.1 Better care from male nurses 3.2 Better care from younger females

#### Theme 1: Social injustice

Participants reported being unfairly treated when accessing primary healthcare facilities. They felt they were discriminated against. These encounters made them feel that their fundamental human rights were infringed upon and that the professional nurses severely transgressed the constitution of the country by mistreating them purely because of their sexual orientation.

**Sub-theme 1.1: Human rights violations:** The participants experienced healthcare disparities when accessing primary healthcare facilities, resulting in violations of their human rights. They stated that despite being in a country where the constitution promotes equality, they felt as if they were still under the apartheid administration, as they did not have access to quality healthcare services and were treated differently because of the colour of their skin and cultural practices. However, within this study, the participants felt that their human rights were violated because of their sexual orientation:

‘My healthcare needs as a gay man are not taken care of in the health institutions. When I access a primary healthcare facility, I feel as if they deny me basic rights and dignity as a person.’ (Participant 1, 33-years-old, male)

‘I feel nurses go out of their way to mistreat me; the shouting, name-calling, and misgendering are uncomfortable. I still experience a lot of challenges that negatively affect me.’ (Participant 6, 33-years-old, female)

**Sub-theme 1.2: Discrimiation:** The participants reported experiencing discriminatory attitudes from professional nurses when accessing primary healthcare facilities:

‘As a lesbian woman living in South Africa, specifically in Gauteng, I experience stigma, discrimination, and bad services when visiting a primary healthcare facility,’ (Participant 12, 27-years-old, female)

‘I kept on telling the nurses what my problem is, but she insisted that maybe it is HIV or AIDS. I feel that was unfair because it does not mean that because I am gay, I have aids.’ (Participant 13, 27-years-old, male)

**Sub-theme 1.3: Compromised confidentiality:** Participants consistently reported breaches of confidentiality, a core tenet of nursing ethics, which further eroded their trust in the healthcare system:

‘They stripped away my confidentiality as if i don’t deserve privacy because this is a free health service,’ (Participant 10, 45-years-old, male)

‘I told her that I am HIV positive and that I feel suicidal, I did not think she would share that with a cleaner of the healthcare facility, she had no right doing that.’ (Participant 4, 41-years-old, male)

#### Theme 2: Professional unpreparedness for inclusive care

The participants shared that they felt that the most relevant factor that contributed to the way professional nurses cared for them was associated with professional nurses not having adequate knowledge about LGBTQI+ persons and their healthcare needs.

**Sub-theme 2.1: Inadequate knowledge, skills and values:** The participants shared that they felt professional nurses lacked the relevant knowledge, skills and values needed to provide inclusive care to LGBTQI+ persons, leading to compromised healthcare services. The following participants provided more insight into this sub-theme:

‘I would say they lack knowledge, or they are just insensitive. I am a transgender woman, but they always misgender me, s.’ (Participant 16, 38-years-old, male)

‘I don’t blame any of these healthcare workers; I think the nursing curriculum is not inclusive of how these healthcare workers should care for others.’ (Participant 15, 44-years-old, male)

**Sub-theme 2.2: Imposing heterosexual norms and values:** The participants revealed that the professional nurses imposed their own beliefs on them. Furthermore, the participants expressed that professional nurses forced their spiritual beliefs and how they wanted the participants to lead their lives, which predominantly stemmed from a heterosexual perspective. The verbatim quotes below give more insight into this sub-theme:

‘“I was asked all sorts of questions, questions that were not relevant and very demeaning, questions such as,” Are you sure you are not already HIV positive? You should rather date women; even if you are HIV negative now, God will still punish you and give you AIDS.’ (Participant 2, 43-years-old, male)

‘“God does not recognise you, that is why you always get sick, I don’t think it is too late for you to change.” That was what the nurse said to me, as if my sexuality gave me low blood.’ (Participant 7, 23-years-old, male).

**Sub-theme 2.3: Lack of humanity in the care they received:** The participants reported that humanity was absent when they accessed primary healthcare facilities in Gauteng, South Africa. The lack of kindness and generosity, combined with the professional nurses’ inability to be sympathetic, made them feel there was a lack of humanity. The verbatim quotes below give a detailed snapshot of the participants’ reality:

‘So if nurses lack humility, how would they respect me as a lesbian? How would they know what to do, or not do? That’s why we’re not included or welcomed when going to a primary healthcare clinic; our needs are not met.’ (Participant 14, 29-years-old, female)

‘I feel the way nurses are unkind and uncaring towards LGBTQI+ people shows they are not accepting us.’ (Participant 5, 25-years-old, male)

#### Theme 3: Experiences of affirmative care

The positive experiences of treatment by the younger generation of professional nurses point the way to better treatment of LGBTQ+ people in Gauteng in the future. The participants revealed that, despite experiencing healthcare disparities when accessing primary healthcare facilities in Gauteng, South Africa, they also reported different experiences when interacting with healthcare workers during their visits. The following verbatim quotes give insight into this theme:

**Sub-theme 3.1: The participants experienced better care from male nurses:** The participants stated that, despite unpleasant experiences in primary healthcare facilities in Gauteng, South Africa, they had positive encounters with male nurses. Participants reported that male nurses demonstrated respect and inclusiveness during their care. The following verbatim quotes give more insight:

‘I pray on the morning of my clinic appointment that a male nurse should examine me; it is almost as if they are different.’ (Participant 9, 29-years-old, male)

‘On that day, I had a soft tissue injury that a male nurse helped me with. He even went to the extent of lending me his phone to call my partner.’ (Participant 14, 29-years-old, female)

**Sub-theme 3.2: The participants experienced better care from younger female nurses:** The participants shared that, despite the undesired healthcare experiences in primary healthcare facilities across the districts, they felt that younger female nurses provided better, more acceptable care. The participants shared the following:

‘Whether it’s genuine or not, younger female nurses are able to treat you better than the older female nurses.’ (Participant 15, 44-years-old, male)

‘That young female nurse stood up for me when those two elderly nurses tried to bully and judge me.’ (Participant 4, 41-years-old, male)

## Discussion

This study highlighted the lack of humanity and distressing experiences of LGBTQI+ persons when accessing primary healthcare facilities in Gauteng, South Africa. Further, the findings indicate primary healthcare nurses did not uphold Watson’s theory of human caring, as LGBTQI+ persons felt that primary healthcare nurses imposed heteronormative norms and values on them, which indicated their inability to provide authentic and non-judgemental care (Watson 2008). In addition, there appeared to be a gap between primary nurses’ practices and the nursing profession’s adherence policies and guidelines, which advocate for justice and non-discrimination, but this seemed to have been breached, as these participants experienced human rights violations (South African Nursing Council [SANC] 2013). The experiences of compromised human rights, such as confidentiality, demonstrated nonadherence to Sections 9 and 14 of the Constitution of South Africa, which advocate equality and privacy (South Africa 1996:ss. 9 & 14). The inability of nurses to provide inclusive healthcare services to LGBTQI+ persons contradicts their scope of practice as outlined in the scope of practice and the global standards of nurses (International Council of Nurses [ICN] 2021; SANC 2020). In conclusion, participants’ experiences indicate that their basic rights, such as confidentiality, privacy, access to care and continuity of care, were breached by primary healthcare nurses (South African Department of Health 1999). The findings provide a clearer lens on the study’s findings and how it breached the core values of nursing and various ethical principles of the Constitution of South Africa.

### Social injustice

Theme 1 hailed from the participants expressing that their human rights were compromised when accessing primary healthcare facilities; thus, the following sub-themes were included in this theme: (1) Human rights violations, (2) discrimination and (3) compromised confidentiality.

### Human rights violations

The participants reported experiencing social injustice when accessing primary healthcare facilities. Luvo and Kangethe (2023) argue that despite legislative frameworks such as the Constitution of South Africa prompting equality and protection of the LGBTQI+ communities, these individuals still experience violations of their human rights, as evidenced by the constant discrimination and homophobic attacks in primary healthcare facilities. Arora, Bhujang and Sivakami (2022) state that the violations of their human rights in healthcare settings are observed through LGBTQI+ people being denied healthcare services and often being discriminated against because of their sexual preferences. Despite this study being conducted within Gauteng, SA, the literature reveals that the violation of the LGBTQI+ persons appears to be a global challenge. As evidenced in Arkansas, the *Medical Ethics and Diversity Act* allowed healthcare workers to refuse care to this community, often leading this population to feel they were denied access to care (Suess Schwend 2020). In Brazil, it was found that individuals who were identified as intersex were often subjected to surgical procedures with the attempt to normalise them, thereby infringing upon their rights to autonomy, bodily integrity and well-being (Leivas et al. 2023).

### Discrimination

The participants felt that professional nurses demonstrated discrimination towards them by creating a heteronormative environment, making incorrect assumptions about their sexual orientation, misgendering and using derogatory language. Badat, Moodley and Paruk (2023) affirm that LGBTQI+ individuals are discriminated against when accessing primary healthcare facilities in Gauteng. Hosseinabadi-Farahani et al. (2023) report that discrimination in the healthcare setting is associated with unfavourable healthcare outcomes linked to physical and mental deficiencies. The impact of discrimination towards LGBTQI + persons contributes to them suffering from high levels of anxiety, depression, substance abuse and even not making use of healthcare facilities in general (Bayram, Weigand & Flatt 2023). Freaney et al. (2024) conclude that when nurses exhibit discriminatory attitudes towards LGBTQI+ persons, it hinders their ability to provide clinically competent healthcare, further promoting uncaring practices. Hendricksen et al. (2022) argue that discrimination in the healthcare setting occurs unconsciously among healthcare workers, as they often lack the relevant skills to provide the desired healthcare services needed by LGBTQI+ persons. Participants in this study also shared that they wished professional nurses would stop discriminating against them. Hendricksen et al. (2022) conclude that discrimination can be addressed only through training that promotes inclusivity for all patients who require healthcare services. Clark and Koenig (2023) allude that diminishing discrimination within a healthcare setting is imperative as discrimination decreases the usage of healthcare services by LGBTQI+ individuals.

### Compromised confidentiality

Hudak, Carmack and Smith (2018) support the phenomenon that healthcare workers often compromise confidentiality when providing care to LGBTQI+ patients by not respecting their preferred pronouns or discussing confidential matters with other healthcare workers, even when there is no need. This often leads to LGBTQI+ persons leaving the healthcare facilities in shame and with the view or decision not to use healthcare facilities in the future (Jamieson et al. 2020). LGBTQI+ persons face challenges regarding confidentiality as the healthcare environment lacks inclusivity of LGBTQI+ individuals, which adversely affects their overall privacy and confidentiality (Beagan et al. 2023). Englund, Basler and Meine (2020) assert that the lack of confidentiality that LGBTQI+ people experience is linked to heteronormative beliefs and ignorance by professional nurses. Pezzella (2023) further suggests that compromised confidentiality within primary healthcare facilities towards LGBTQI+ persons is a result of discrimination, stigma and non-inclusive healthcare policies. The lack of confidentiality towards LGBTQI+ persons reflects a breach of their human rights and the core fundamental of the nursing profession, which often results in mistrust and compromised communication between the healthcare workers and the LGBTQI+ persons (Goldberg, Rosenburg & Watson 2018).

### Professional nurses’ unpreparedness for inclusive care

This theme depicted that professional nurses were not empowered to provide care to the participants, and the following sub-themes were established: (1) Inadequate knowledge, skills and values, (2) imposing heterosexual norms and values and (3) lack of humanity.

### Inadequate knowledge, skills and values

Bloompott et al. (2023) state that the existing nursing curriculum frequently exhibits insufficient incorporation of LGBTQI considerations, which profoundly affects the quality of care provided to this population. In addition, empirical studies suggest that nursing education often neglects critical subjects pertinent to LGBTQI health, resulting in a deficiency in knowledge and readiness among nursing practitioners (Klepper et al. 2023). Furthermore, the lack of representation of LGBTQI+ topics within nursing curricula fosters an environment characterised by invisibility and stigma, which may discourage LGBTQI individuals from pursuing essential healthcare services (Cassidy et al. 2023; Day, Snyder & Dennis Flores 2023). A recent study in the South African context found that professional nurses’ lack of understanding of and training in the LGBTQI+ sexual and reproductive healthcare services compromised access by LGBTQI+ individuals to primary healthcare facilities (Seretlo & Mokgatle 2023). Englund et al. (2020) conclude that the lack of LGBTQI+ content within the nursing curriculum accelerates health disparities and promotes a healthcare environment that focuses on the healthcare needs of heterosexual individuals.

### Imposing heterosexual norms and values

Öcalan and Hicdurmaz (2024) assert that the imposition of personal beliefs by professional nurses upon LGBTQI+ individuals may result in substantial healthcare disparities and a deterioration in the quality of care provided. The reality of professional nurses imposing their personal beliefs and cultures on LGBTQI+ persons emanates from the homophobic and transphobic practices within the nursing environment (Westwood, James & Hafford-Letchfield 2023). A study in Turkey revealed that professional nurses impose their own beliefs and biases on LGBTQI+ persons, as they believe that these individuals disrupt social norms (Aslan & Paslı Gürdoğan 2024). Ghio, Malsch and McGuigan (2025) allude that the phenomenon of nurses imposing their personal beliefs on LGBTQI+ persons raises significant concerns, particularly as healthcare settings have traditionally favoured heterosexual paradigms, thereby marginalising LGBTQ+ identities to a significant extent. Similar findings in Canada concluded that, aside from professional nurses imposing their personal beliefs on LGBTQI+ individuals, nurses often offer false support rather than genuine inclusive support (Haghiri-Vijeh 2023).

### Lack of humanity

Arora et al. (2022) found that LGBTQI+ persons often experience a lack of humanity in healthcare environments, which is the result of constant discrimination, homophobic attacks and nurse bias. Similar studies concur that in healthcare settings, the lack of humanity towards LGBTQ+ persons is often experienced through disrespect, insufficient care, rudeness, bullying and insensitivity from professional nurses (Stein et al. 2023). The lack of humility in professional nurses when caring for LGBTQI+ persons promotes emotional distress among this population, which erodes trust in healthcare professionals and intensifies unmet needs (Allison et al. 2024). A study by Nair et al. (2021) found that barriers, such as professional nurses’ deficient knowledge of the LGBTQI+ community, contribute to a gap in their ability to provide patient-centred, humble care. Pearce and Di Lorito (2023) state that professional nurses have voiced that they lack the relevant skills, knowledge and values required to provide quality care; individualised care that is centred around humanity towards LGBTQI+ persons and other marginalised populations.

### Experience of affirmative care

This theme unveiled some positive findings which the participants experienced when accessing primary healthcare facilities, as follows: (1) Quality care from male nurses and (2) quality care from younger female nurses.

### Quality care from male nurses

Callan, Corbally and McElvaney (2023) and Pratt-Chapman et al. (2022) concur that despite the healthcare disparities experienced by LGBTQI+ persons when accessing healthcare facilities, male nurses appear to be providing better care and understanding to this population. Duckett and Ruud (2019) affirm that male nurses are more inclined to provide inclusive healthcare to LGBTQI+ persons and other marginalised groups. The literature provides evidence that male nurses in the nursing profession provide satisfactory healthcare services to all patients and communities, as they appear receptive to diverse cultural dynamics (Budu et al. 2019). Margolies and Brown (2019) assert that it is imperative for all professional nurses to culturally promote competent, caring practices when providing care to LGBTQI+ persons to diminish health disparities and health-related risks. Sirufo et al. (2022) argue that despite the positive experiences LGBTQI+ persons have had with male nurses, healthcare practices can only be improved through the inclusion of approved training and educational content focused on the LGBTQI+ community. Furthermore, Cottingham, Johnson and Taylor (2016) contribute that some men within the nursing profession might still be providing care to LGBTQI+ persons that is based on stereotypes and homophobia, which are promoted through societal heteronormative practices.

### Quality care from younger female nurses

Young female nursing professionals frequently display an inclination towards self-reflection and the modification of their communicative approaches, a practice deemed crucial for effectively catering to the distinctive requirements of LGBTQI+ patients (Reeves et al. 2024). Landry and Kensler (2019) state that emerging young female nurses can deliver enhanced healthcare services to LGBTQI patients by understanding health inequities, refining communication practices and fostering inclusive environments informed by culturally sensitive protocols outlined in the document. Similarly, a study in Portugal found that young female nurses can provide better care to LGBTQI+ persons because of their positive attitudes and acceptance of all marginalised individuals (Gomes et al. 2023). Despite the literature indicating the positive contributions young females make to caring for LGBTQI+ persons, Webb and Zablocki (2023) argue that there is still a need for all healthcare providers, including young female nurses, to be empowered with inclusive care skills to promote culturally competent care for LGBTQI+ persons.

### Strengths of this study

The secondary researcher served as the supervisor during data collection, thereby enhancing the credibility of the research findings. To further promote credibility, data analysis was conducted by an independent coder. In addition, the primary researcher remained in the field for approximately 6 months; this allowed the researchers to explore the phenomenon in depth and make sense of LGBTQI+ persons’ experiences when accessing primary healthcare facilities in Gauteng, South Africa.

### Limitations of this study

The study lacked methodological triangulation, as the researchers focused uniquely on face-to-face, unstructured interviews with individuals, limiting the possibility of verifying the experiences of other LGBTQI+ persons when accessing primary healthcare facilities in Gauteng, South Africa.

#### Implications for nursing practice

The findings of this study unveiled the devastating experiences of care that LGBTQI+ persons experience within primary healthcare facilities. Nursing institutions, not limited to primary healthcare facilities, can utilise the findings of this study to conduct their own assessment of their status in providing care to LGBTQI+ persons. Based on these findings, they can develop operational strategies that are sustainable to promote inclusivity of the healthcare services provided within healthcare facilities. In addition, these institutions may also incorporate diversity and sensitivity into service training for all healthcare workers or develop inclusive operational policy guidelines. In conclusion, McNamara and Ng (2016) state that to promote inclusive care to LGBTQI+ persons, healthcare nurses need to be clinically and culturally competent.

#### Implications for nursing education

As nursing education is governed by various legislative frameworks and, given that this study’s findings emerged from the Gauteng province, there is a clear call for the inclusion of culturally competent, LGBTQI+-affirming care in nursing curricula. This advocacy is essential for preparing future nurses to deliver equitable healthcare. In addition, curriculum reform should include modules on gender identity, sexual orientation, communication and ethics in caring for sexual minorities.

#### Implications for further research

Further research is needed to investigate the systemic factors behind this disconnect. This could include collaboration with medico-legal experts to analyse reports or legal cases filed by LGBTQI+ individuals concerning discrimination or abuse within healthcare settings. In addition, mixed-methods approaches, or interventional studies, may be conducted to evaluate the impact of inclusive training for professional nurses providing care to LGBTQI+ persons in primary healthcare facilities in Gauteng, South Africa.

## Conclusion

In conclusion, while LGBTQI+ persons continue to face systemic health disparities within primary healthcare facilities, the emerging evidence of affirmative care from younger and male nurses signals the potential for generational transformation. This underscores the urgency for education, policy reform and institutional accountability to realise equitable healthcare for all.
